# Sublobar resection utilizing near-infrared thoracoscopy with intravenous indocyanine green for intralobar pulmonary sequestration: a case report and literature review

**DOI:** 10.1186/s40792-023-01758-w

**Published:** 2023-10-07

**Authors:** Chiaki Kanno, Yujin Kudo, Ryosuke Amemiya, Jun Matsubayashi, Hideyuki Furumoto, Satoshi Takahashi, Sachio Maehara, Masaru Hagiwara, Masatoshi Kakihana, Toshitaka Nagao, Tatsuo Ohira, Norihiko Ikeda

**Affiliations:** 1 Department of Surgery, Tokyo Medical University, 6-7-1 Nishishinjuku, Shinjuku-Ku, Tokyo, 160-0023 Japan; 2 Department of Anatomic Pathology, Tokyo Medical University, Tokyo, Japan

**Keywords:** Pulmonary sequestration, Pryce classification, Indocyanine green (ICG), Video-assisted thoracic surgery (VATS)

## Abstract

**Background:**

Pulmonary sequestration is a rare pulmonary malformation, with intralobar pulmonary sequestration being the most common subtype. Lobectomy has generally been performed for its treatment, owing to unclear boundaries of the lesion. However, recent reports have introduced lung resection using intravenous indocyanine green (ICG) as a treatment for pulmonary sequestrations.

**Case description:**

A 34-year-old woman presented with chest pain, and enhanced chest computed tomography (CT) displayed a solid mass of 4.5 × 3.1 cm in the right S10 area. An aberrant artery was found running from the celiac artery through the diaphragm to the thoracic cavity. The patient was diagnosed as having pulmonary sequestration Pryce type III, and surgical resection was performed. Intrathoracic findings demonstrated that the precise area of the pulmonary sequestration could not be clearly identified, and a 5-mm aberrant artery was present in the pulmonary ligament. Following the separation of the aberrant artery, intravenous injection of ICG clearly delineated the border between the normal lung tissue and the pulmonary sequestration. Wedge resection was then performed without any postoperative events, and the pathological diagnosis was also pulmonary sequestration.

**Conclusions:**

We herein reported a case of a patient who underwent sublobar resection for intrapulmonary sequestration using intravenous ICG injection, together with a literature review. Our case suggests that a comprehensive understanding of abnormal vessels and pulmonary vasculature in pulmonary resection for intrapulmonary sequestrations, complemented with the use of ICG, might potentially avoid unnecessary pulmonary resection and enable sublobar surgical resection.

## Introduction

Pulmonary sequestration is a rare congenital malformation of the respiratory tract, which involves a nonfunctioning portion of the lung parenchyma without normal communication with the bronchus, and is connected to an aberrant vessel arising from the systemic circulation [1]. The disease is difficult to diagnose owing to its unusual clinical features and anatomical variations. Pulmonary sequestration can be divided into 2 categories based on the correlation between the sequestration and the normal lungs, namely, intralobular pulmonary sequestration (ILS) and extralobular pulmonary sequestration (ELS). The more frequent type is ILS, which is encased in the pleura of the same lung and is observed in the basal left lower lobe in two-thirds of cases [2]. The gold standard treatment is resection of the sequestered lung. Until recently, lobectomy was the principal surgical procedure, as the demarcation of the ILS is often unclear. However, there have been some reports of sublobar resection, such as segmentectomy or wedge resection, using intravenous indocyanine green (ICG) to delineate the ILS lesion [3–5]. Herein, we present a case of a patient in whom ILS was successfully identified using ICG, and was safely resected without excess or insufficient tissue resection.

## Case presentation

**Fig. 1:**
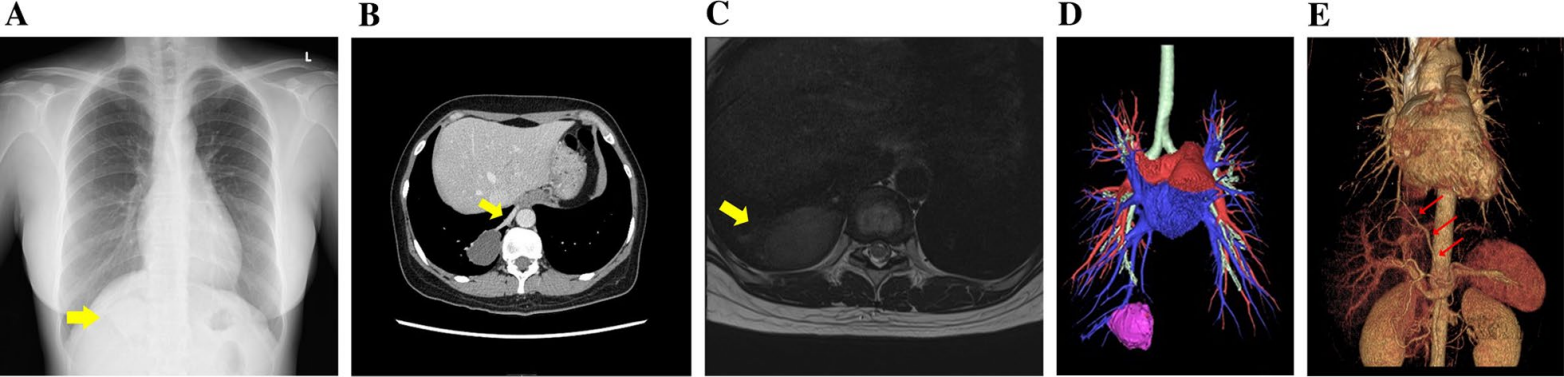
**A** Chest X-ray displayed a solid nodule (yellow arrow) in the right lower lung field. **B** Enhanced chest computed tomography displayed a solid mass of 4.5 × 3.1 cm in the right S10 area, accompanied by an aberrant artery (yellow arrow) supplying blood to the mass. **C** Chest magnetic resonance imaging displayed low intensity on T2-weighed image in the mass area (yellow arrow). **D** Three-dimensional reconstruction image shows the involvement of pulmonary veins (blue) in the mass (pink), while neither the pulmonary artery (red) nor the bronchus (light green) was involved. **E** Three-dimensional reconstruction image shows that the aberrant vessel (red arrows) inflowing into the mass is found to branch off from the celiac artery

A 34-year-old woman presented with chest pain that had started on that day, and abnormal shadows were displayed on chest computed tomography (CT). She had no past medical history and no smoking history. On physical examination, her vital signs were within the normal range. No significant abnormalities were detected in laboratory blood tests. Chest X-ray displayed a solid nodule in the right lower lung field (Fig. 1A). Enhanced chest CT displayed a solid mass of 4.5 × 3.1 cm in the right S10 area (Fig. 1B). An aberrant artery was found running from the celiac artery through the diaphragm to the thoracic cavity, which was located along the border of the mass. The mass lacked continuity with the bronchus. Chest magnetic resonance imaging displayed high intensity on T1-weighted images and low intensity on T2-weighted images in the area of the mass (Fig. 1C). Three-dimensional (3D) reconstruction images showed aberrant arterial inflow into the mass, and the involvement of pulmonary veins in the mass, but no involvement of pulmonary arteries or bronchioles (Fig. 1D, and E). Therefore, the patient was diagnosed as having pulmonary sequestration Pryce type III, and surgical resection was performed.

**Fig. 2:**
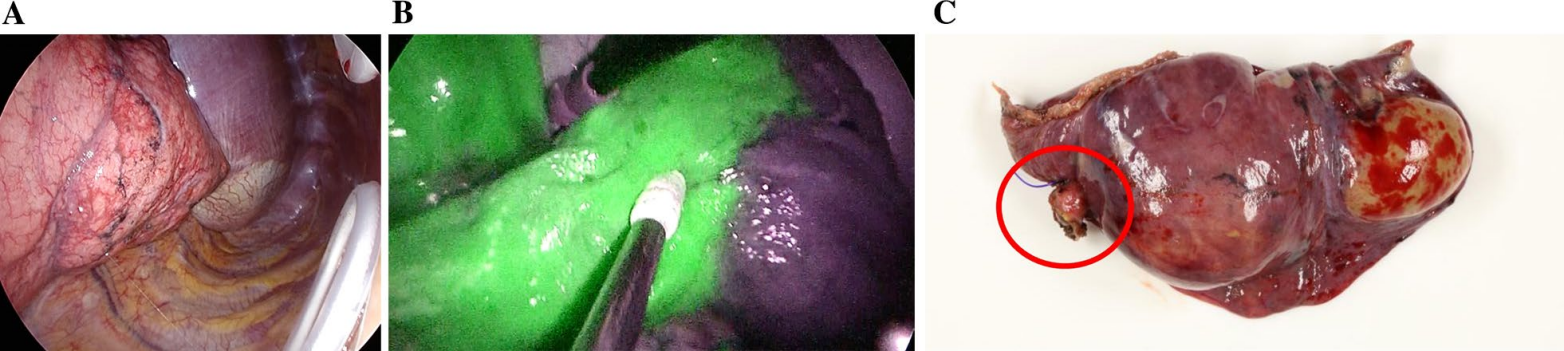
**A** Intrathoracic findings using white light indicated that hypervascularity in the pleura of the right lower lobe was present, but the borders between the mass and normal lung were unclear. **B** Intrathoracic findings using near-infrared light after intravenous injection of indocyanine green demonstrated that the border between the normal lung (stained in green) and the mass (not stained) was clearly demarcated. **C** Pathological findings showed that the resected lung specimen had a 4.5 × 3.5 × 2.5 cm cystic lesion, which contained brown fluid inside. The ligated aberrant artery is indicated with a red circle

The patient was put under general anesthesia and in the left lateral decubitus position, and wedge resection of the right lower lobe and dissection of the abnormal vessels were performed by four-port video-assisted thoracoscopic surgery (VATS). A 2-cm port at the seventh intercostal midaxillary line and a 0.7 cm port at the sixth intercostal posterior axillary line were used as the surgeon’s ports. A 1.2-cm port for the camera was placed at the fifth intercostal anterior axillary line, and a 1.5 cm port for the assistant surgeon at the sixth intercostal anterior axillary line. Intrathoracic findings demonstrated the presence of increased capillaries in the pleura of the right lower lobe, where the pulmonary sequestration was suspected to be located, but the borders could not be clearly identified (Fig. 2A). Furthermore, a 5-mm aberrant artery was present in the pulmonary ligament that penetrated the diaphragm and drained into the pulmonary sequestration in the right lower lobe. No adhesion or pleural effusion was observed.

After dissecting the tissue surrounding the aberrant artery, the proximal side of the abnormal artery was stab-ligated with a 4-0 prolene suture (Ethicon, Japan), and the peripheral side was coagulated and sectioned with an energy device. Five mg of ICG (DAIICHI SANKYO CO., LTD, Japan) was intravenously injected, and near-infrared light thoracoscopy (VISERA ELITE II, OLYMPUS CORPORATION, Japan) clearly demarcated the border between the normal lungs and the pulmonary sequestration (Fig. 2B).Wedge resection of the right lower lobe was performed with the observed boundary as an indicator. The patient’s postoperative course was uneventful, with no adverse events. Six months after the surgical resection, the patient was being followed up in the outpatient clinic, without the development of an aneurysm.

Grossly, a 4.5 × 3.5 × 2.5 cm cyst was observed in the resected lung, which contained brown fluid inside (Fig. 2C). Histologically, pulmonary tissue was observed in the mass, and the inner surface of the cyst was coated with multiciliate epithelia. The aberrant artery was an elastic vessel, with a fibrous and mildly thickened internal lumen. The lesion was concluded to be a pulmonary sequestration, which was consistent with the preoperative diagnosis.

## Discussion

In the present case, VATS with ICG-based infrared light was successfully used to clearly demarcate the border of the sequestrated lung in a patient with intralobar pulmonary sequestration, and minimal resection was performed to avoid unnecessary pulmonary resection. Importantly, we were able to accurately diagnose the feeding artery of the pulmonary sequestration by precisely delineating the association between the aberrant vessels, pulmonary arteries, and pulmonary veins utilizing 3D imaging reconstruction.

Pulmonary sequestration is categorized as ILS or ELS. ILS, which accounts for about 83.9% of pulmonary sequestrations, consists of a common visceral pleura shared with adjacent lung tissue, whereas ELS, which accounts for about 16.1% of sequestrations, is encapsulated by an independent visceral pleura [1]. Regarding symptoms, ILS is often associated with repeated pneumonia and hemoptysis. ELS, however, is generally asymptomatic or is discovered incidentally during routine physical examinations. ELS is more frequently accompanied with other congenital anomalies than ILS. It was reported that approximately 50–60% of patients with ILS have another congenital abnormality, of which congenital diaphragmatic hernia is the most common [2, 6]. Although our present patient was diagnosed as having ILS, she had not experienced any episodes of pneumonia or hemoptysis previously, and it remains unclear whether the chest pain was associated with this disease. From the patient’s intrathoracic findings, it was difficult to demarcate the border of the surface pulmonary sequestration by thoracoscopic imaging with white light.

Pulmonary sequestration was described by Pryce in 1946, who classified ILS into 3 types [7, 8]. In type I, an aberrant artery flows through part of the normal lung but not through the sequestration; in type II, the aberrant artery flows through both the normal lung and the sequestration; and in type III, the aberrant artery flows only to the sequestration. Subsequently, however, it was found that type I was very different from a pulmonary sequestration, and Pryce reported that what was originally considered to be type I ILS was actually an anomalous systemic arterial supply to the lung. The present patient had a Pryce type III sequestration, in which the aberrant artery from the celiac artery flowed only to the sequestration. Savic et al. reported that 57% of intrapulmonary sequestrations were located on the left side, and 43% were on the right side [2]. Furthermore, 74% of the aberrant arteries branched from the descending aorta, and only 1% branched from the celiac artery, such as in the present patient.

Recently, the use of near-infrared thoracoscopy with intravenous ICG has been reported for the identification of the borders of pulmonary sequestrations [3–5, 9–16]. In the 15 reported cases to date, including this case, of patients with a pulmonary sequestration who underwent surgical resection with intravenous ICG, sublobar resection, such as segmentectomy or wedge resection were performed, instead of lobectomy (Table 1). We retrieved relevant or referenced articles on Pubmed^®^ using the keywords “pulmonary sequestration,” “indocyanine green,” and “near-infrared light”. Out of 15 cases that underwent sublobar resections with intravenous ICG, 3 cases involved segmentectomy, while 12 cases involved wedge resection. The majority of the cases with wedge resection exhibited no inflow from the pulmonary artery to pulmonary sequestrations, no communication between normal bronchioles and pulmonary sequestrations, and no inflow from abnormal vessels to the normal lung. It was suggested that in cases, where the area of pulmonary sequestrations is clearly delineated by intravenous ICG through the treatment of abnormal vessels, wedge resection becomes feasible if it is located in the peripheral lung.

**Table 1:** Summary of the 15 patients who underwent surgical resection of a pulmonary sequestration utilizing intravenous indocyanine green

Age	Sex	Location of PS	Characteristics of PS	Origin of aberrant vessel	Inflow of aberrant vessel into PS	Inflow of pulmonary artery into PS	Connection between normal bronchus and PS	Surgical procedure	Approach	Reference
33	M	Left	Mass	Descending aorta	No	No	No	Wedge resection	Thoracotomy	4
38	F	Right	Cystic mass	Descending aorta	Unknown	No	No	Wedge resection	VATS	4
24	F	Right	Large pulmonary consolidation	Celiac artery	Unknown	Unknown	No	Segmentectomy	VATS	4
42	F	Right	Multiple cystic lesions	Descending aorta	No	No	No	Wedge resection	VATS	5
44	M	Right	Multiple cystic and solid masses	Celiac trunk	No	No	No	Wedge resection	Thoracotomy	10
53	F	Right	Mass	Abdominal aorta	No	Unknown	No	Wedge resection	Thoracotomy	10
33	M	Left	Overinflated area, multiple cystic lesions	Descending aorta	No	No	No	Segmentectomy	VATS	3
41	F	Right	Mass	Right renal artery	No	No	No	Wedge resection	VATS	11
39	F	Left	Overinflated area, multiple cystic lesions	Thoracic aorta	No	No	No	Wedge resection	RATS	13
21	F	Right	Hyperlucent area	Descending aorta	No	No	No	Wedge resection	RATS	12
25	F	Left	Nodular consolidation	Descending aorta	No	No	Yes	Wedge resection	Uniportal VATS	9
56	F	Left	Multiple cystic lesions	Abdominal aorta	No	No	No	Wedge resection	VATS	14
68	F	Right	Unknown	Descending aorta	Unknown	No	Unknown	Segmentectomy	Thoracotomy	15
38	M	Right	Cyst	Celiac artery	No	No	No	Wedge resection	VATS	16
34	F	Right	Cyst	Celiac artery	No	No	No	Wedge resection	VATS	This case

*M* male, *F* female, *PS* pulmonary sequestration, *VATS* video-assisted thoracic surgery, *RATS* robotic-assisted thoracic surgery

For demarcation of the border between the pulmonary sequestration and the normal lung tissue by intravenous ICG, it is crucial to understand the association between the aberrant vessels, pulmonary arteries, and pulmonary sequestration from the 3D images. A previous study demonstrated that 3D-CT is very useful, and Synapse Vincent (FUJIFILM Corporation, Japan) was utilized to generate the 3D images of the cases [17, 18]. Consequently, the associations among the pulmonary sequestration and the aberrant vessels could be clarified, and an appropriate surgical strategy could be planned. In cases, such as Pryce type II of ILS, in which the blood flow from the aberrant vessels and the pulmonary artery flow into the pulmonary sequestration, it is not possible to demarcate the area using intravenous ICG unless the blood flow from the associated pulmonary artery is blocked. However, as in the present patient, it is possible to perform sublobar resection only by blocking the flow from the aberrant vessel, if it is identified that the flow is only from the aberrant vessel. Therefore, the relationship between aberrant vessels, pulmonary arteries, and bronchus in pulmonary sequestration can be better elucidated with the use of 3D imaging, which is important for determining the appropriate surgical procedure.

## Conclusions

We herein reported a case of a patient who underwent sublobar resection for intrapulmonary sequestration Pryce type III by near-infrared thoracoscopy together with intravenous ICG injection. Based on our present case and review of the literature, it is suggested that for the resection of intrapulmonary sequestrations, a comprehensive understanding of abnormal vessels and the pulmonary vasculature, complemented with the use of ICG, might potentially avoid unnecessary pulmonary resections and enable sublobar resection.

## Data Availability

All data supporting the conclusions of this article are included within the published article.

## Abbreviations

ICG: Indocyanine green
VATS: Video assisted thoracic surgery
ILS: Intralobular pulmonary sequestration
ELS: Extralobular pulmonary sequestration
CT: Computed tomography
MRI: Chest magnetic resonance imaging
